# Mitochonic acid 5 alleviates amyotrophic lateral sclerosis phenotypes via mitochondrial augmentation

**DOI:** 10.1172/jci.insight.200761

**Published:** 2026-09-22

**Authors:** Yoshitsugu Oikawa, Yuhan Luo, Naoki Suzuki, Tomoko Kasahara, Yoshiyasu Tongu, Yuki Yoshida, Tsukasa Tominari, Shogo Tanabe, Yoshiko Suto, Hitomi Kashiwagi, Saki Saito, Kensuke Ikeda, Chitose Suzuki, Arata Kuranaga, Tetsuya Akiyama, Satoru Morimoto, Yoshitsugu Aoki, Rieko Muramatsu, Tomoyoshi Soga, Masashi Aoki, Hideyuki Okano, Tetsuhiro Tanaka, Takaaki Abe, Erina Kuranaga, Takafumi Toyohara

**Affiliations:** 1 Department of Medical Science, Tohoku University Graduate School of Biomedical Engineering, Sendai, Japan.; 2 Department of Pediatrics, Tohoku University Graduate School of Medicine, Sendai, Japan.; 3 Laboratory for Histogenetic Dynamics, Graduate School of Life Sciences, Tohoku University, Sendai, Japan.; 4 Department of Neurology and; 5 Department of Rehabilitation Medicine, Tohoku University Graduate School of Medicine, Sendai, Japan.; 6 Department of Molecular Therapy and; 7 Department of Molecular Pharmacology, National Institute of Neuroscience, National Center of Neurology and Psychiatry, Tokyo, Japan.; 8 Laboratory for Histogenetic Dynamics, Graduate School of Pharmaceutical Sciences, Kyoto University, Kyoto, Japan.; 9 Keio University Regenerative Medicine Research Center, Kawasaki, Japan.; 10 Division of Neurodegenerative Disease Research, Tokyo Metropolitan Institute for Geriatrics and Gerontology, Tokyo, Japan.; 11 Human Biology-Microbiome-Quantum Research Center, Keio University, Tsuruoka, Yamagata, Japan.; 12 Department of Nephrology and Hypertension, Tohoku University Hospital, Sendai, Japan.; 13 Department of Clinical Biology and Hormonal Regulation, Tohoku University Graduate School of Medicine, Sendai, Japan.; 14 Division of Bioengineering for Renal and Circulatory Regeneration, Tohoku University Graduate School of Biomedical Engineering, Sendai, Japan.

**Keywords:** Metabolism, Neuroscience, ALS, Biomarkers, Drug therapy

## Abstract

Amyotrophic lateral sclerosis (ALS) is a fatal neurodegenerative disease that urgently requires effective treatment. Mitochondrial dysfunction underlies ALS pathology and represents a potential therapeutic target. Here, we demonstrated the therapeutic potential of mitochonic acid 5 (MA-5), a novel mitochondria-targeted compound that ameliorated ALS phenotypes by enhancing mitochondrial function. In a *Drosophila* ALS model expressing a mutant human *SOD1* (G85R), MA-5 significantly improved locomotor activity, with a trend toward restoration of mitochondrial integrity. In skin fibroblasts derived from ALS patients and motor neurons derived from induced pluripotent stem cells, MA-5 restored ATP production and increased mitochondrial motility. Multiomics analyses suggested that MA-5 modulated mitochondria-linked gene expression and downregulated the glycerophosphate shuttle, contributing to mitochondrial reactive oxygen species production. Transcriptomic analysis identified *C7orf31* as a potential marker for monitoring the therapeutic effects of MA-5 and diagnosing ALS subtypes. These findings support MA-5 as a promising therapeutic candidate for ALS and propose *C7orf31* as a potential biomarker for treatment monitoring and for disease subtyping.

## Abstract 2

MA-5, a mitochondria-targeted compound, improved mitochondrial function and ameliorated amyotrophic lateral sclerosis phenotypes in Drosophila, patient-derived fibroblasts, and iPSC-derived motor neurons.

## Graphical abstract

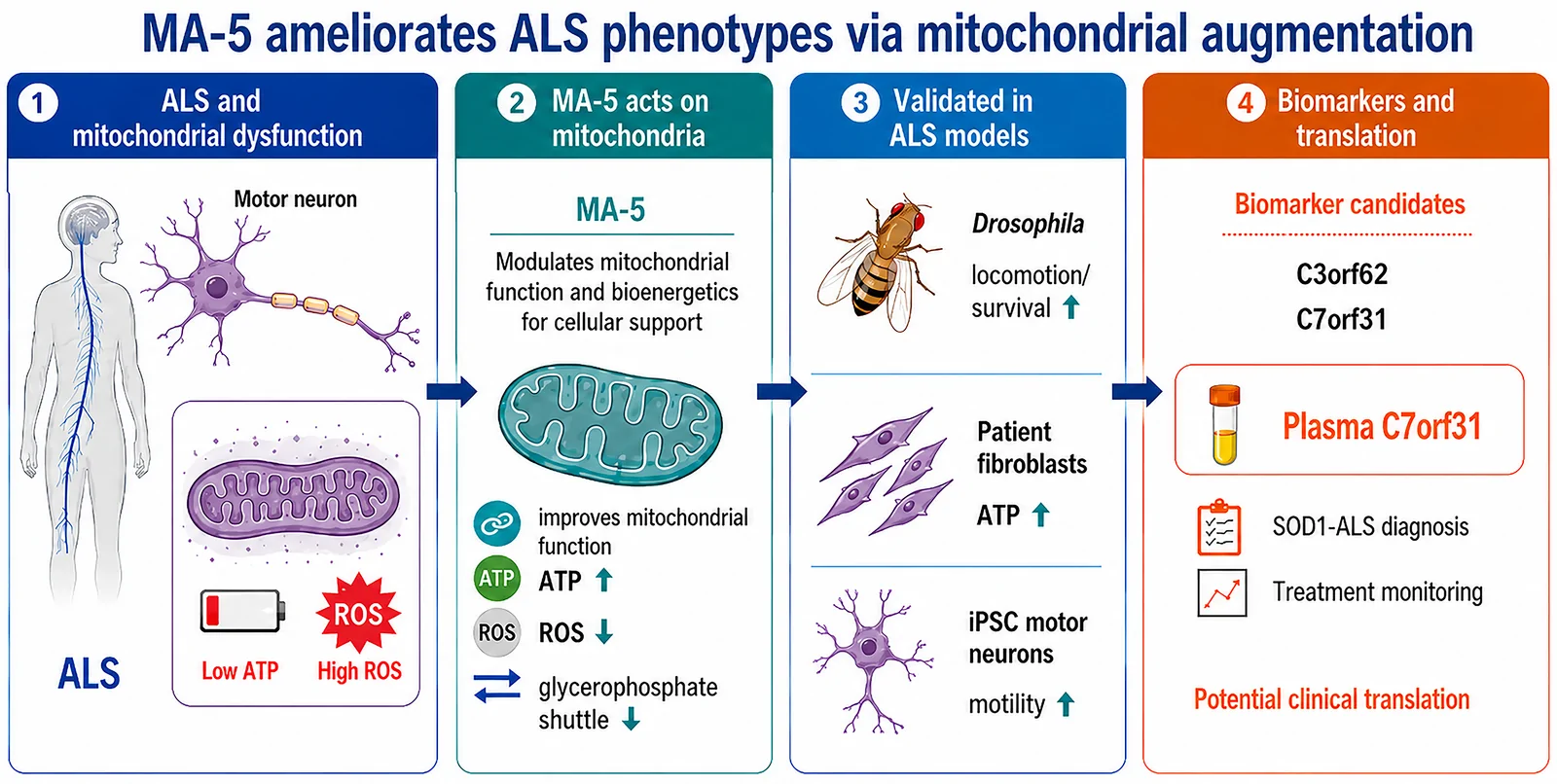

## Introduction

Amyotrophic lateral sclerosis (ALS) is the most common type of motor neuron disease and is characterized by the selective degeneration of motor neurons. The prevalence of ALS is 4–6 per 100,000 individuals, with variations observed between cohorts (1, 2). Approximately 10% of ALS cases are familial, with more than 20 genes implicated in disease onset, including *superoxide dismutase 1* (*SOD1*), *TAR DNA-binding protein* (*TARDBP*; *TDP-43*), *fused in sarcoma* (*FUS*), and *C9orf72* (3). The median survival time is approximately 3 years from symptom onset, with respiratory failure being the primary cause of death (4). Despite its incurable and fatal nature, only a few drugs have been approved by the FDA — riluzole, edaravone, and tofersen (5). Among these, riluzole is the most widely used, extending survival by only a few months (6). Although extensive research has been conducted to identify novel therapies and clinical markers for ALS, significant breakthroughs are urgently needed.

Mitochondria play a pivotal role in ALS pathogenesis. Neurons, which have high metabolic demands, require substantial energy, with the brain consuming approximately 20% of the body’s ATP production (1). Mitochondria generate ATP through oxidative phosphorylation and regulate critical cellular processes, including apoptosis, calcium homeostasis, and neurotransmitter release (7). Given its importance, mitochondrial dysfunction is closely associated with the onset and progression of ALS (8, 9). Several familial ALS-associated genes, including *SOD1*, *FUS*, *TARDBP*, and *C9orf72*, have been linked to mitochondrial dysfunction (10). Mutations in *CHCHD10*, a gene encoding a mitochondrial protein, have been implicated in ALS onset (11). Pathological studies have identified dense clusters of mitochondria in the anterior horn of the lumbar spinal cord in patients with ALS (12), as well as swollen presynaptic mitochondria in their motor neurons (13). Moreover, mitochondrial dysfunction extends beyond neurons to muscle fibers in patients with ALS, potentially contributing to the disease’s etiology (14, 15). Collectively, these findings underscore the critical role of mitochondria in ALS pathogenesis.

Despite the clear involvement of mitochondrial dysfunction in ALS, no effective treatment targeting mitochondrial abnormalities has been developed. Mitochondrial dysfunction contributes to oxidative stress, ATP depletion, and ultimately cell death. Current approaches to mitigate mitochondrial dysfunction are broadly categorized into 3 strategies: (a) enhancing energy efficiency via gene regulators, such as AMP-activated protein kinase (AMPK) and peroxisome proliferator–activated receptor (PPAR); (b) reducing reactive oxygen species (ROS) production using antioxidant quinones, such as coenzyme Q_10_ and idebenone; and (c) modulating mitochondrial dynamics through fission, fusion, and mitophagy (16). However, no drugs based on these mechanisms have yet been established, and many remain in clinical trials to improve mitochondrial functions.

We recently reported a novel therapeutic compound, mitochonic acid 5 (MA-5), which enhances mitochondrial function without inducing ROS production and has completed phase I clinical trials (jRCT2031210495, Japan Registry of Clinical Trials). MA-5 targets mitofilin/MIC60, facilitating ATP synthase oligomerization and supercomplex formation while preserving mitochondrial respiration and glycolysis (17, 18). MA-5 has demonstrated efficacy in treating mitochondria-linked conditions, including hereditary mitochondrial diseases, acute kidney injury (18, 19), and inclusion body myositis (20).

In the present study, we investigated the therapeutic potential of MA-5 in ALS and elucidated its mechanism of action. Additionally, using patient-derived induced pluripotent stem cells (iPSCs), we identified C7orf31 as a biomarker for distinguishing ALS subtypes and monitoring the therapeutic effects of MA-5. Our findings suggest that MA-5 is a promising therapeutic candidate for ALS and highlight the utility of patient-derived iPSCs as a platform for modeling ALS and evaluating treatments.

## Results

### MA-5 ameliorated ALS phenotypes in Drosophila by improving mitochondrial function.

**Figure 1:**
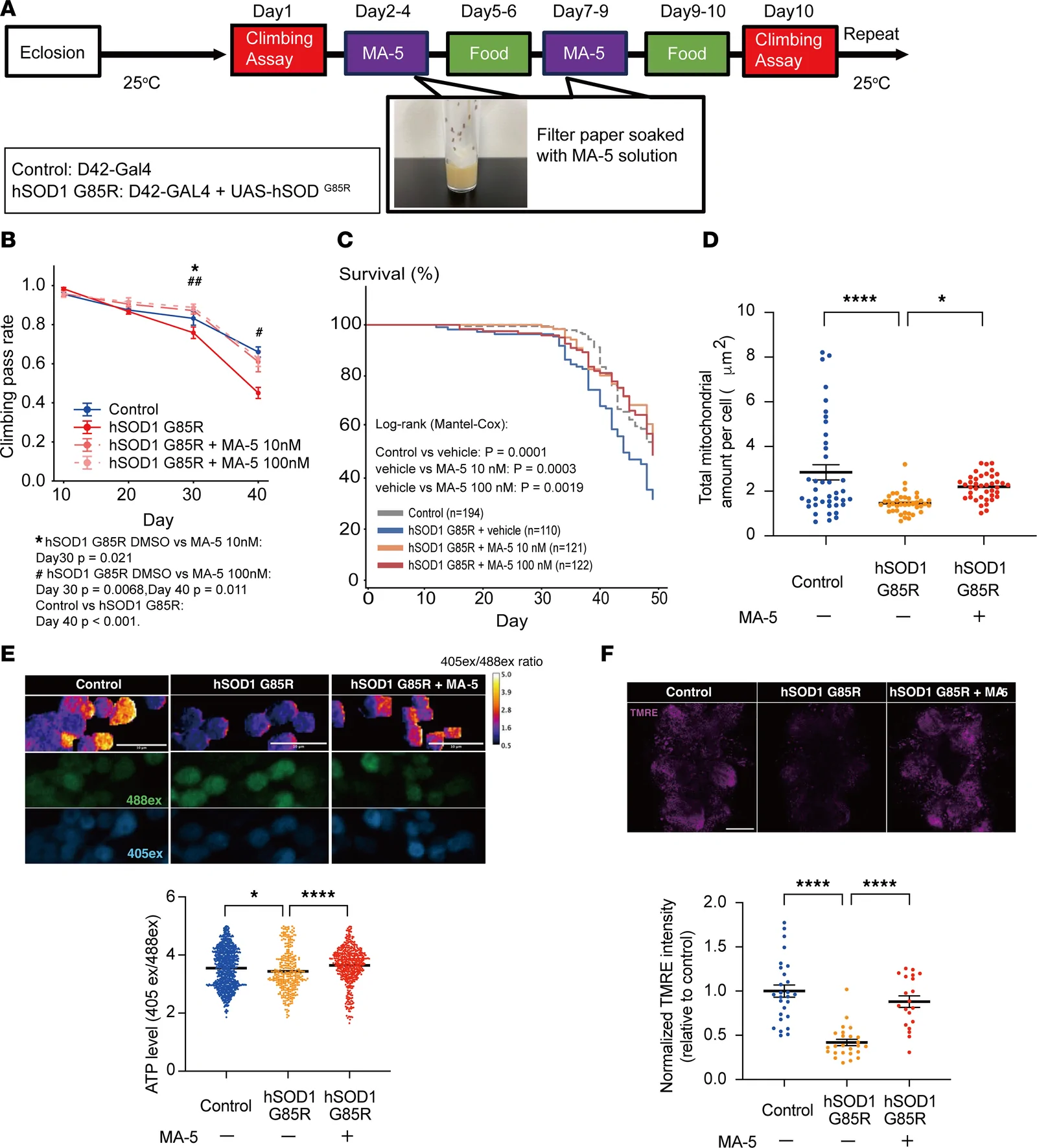
MA-5 ameliorates motor dysfunction and mitochondrial abnormalities in *Drosophila* ALS models. (**A**) Experimental design of MA-5 administration in *Drosophila*. MA-5 was delivered through filter paper soaked with MA-5–containing sucrose solution. (**B**) Longitudinal climbing performance in control flies and hSOD1^G85R^ ALS-model flies with or without MA-5 (10 or 100 nM). Climbing performance was evaluated from day 10 to day 40 (*n* = 8 vials/group at days 10 and 20; *n* = 11 vials/group at days 30 and 40) and analyzed by 2-way repeated-measures ANOVA with Bonferroni correction. (**C**) Kaplan-Meier survival analysis of adult-onset hSOD1G^85R^ ALS-model flies with or without MA-5 (control, *n* = 194; vehicle, *n* = 110; MA-5 10 nM, *n* = 121; MA-5 100 nM, *n* = 122). Survival curves were compared by log-rank (Mantel-Cox) test. (**D**) Total ATP5A-positive mitochondrial area per motor neuron (*n* = 40 neurons from 4 flies/group), analyzed by 1-way ANOVA followed by Tukey’s multiple-comparison test. (**E**) Intracellular ATP levels in motor neurons measured with the ratiometric biosensor QUEEN-7μ (52) (control, *n* = 3 flies; hSOD1^G85R^, *n* = 4; hSOD1^G85R^ + MA-5, *n* = 4), analyzed by 1-way ANOVA followed by Tukey’s honestly significant difference (HSD) post hoc test. Scale bars: 10 μm. (**F**) Mitochondrial membrane potential assessed by TMRE staining in larval ventral nerve cords (control, *n* = 27; hSOD1^G85R^, *n* = 25; hSOD1^G85R^ + MA-5, *n* = 20; 5 independent experiments/group), analyzed by 1-way ANOVA followed by Tukey’s HSD post hoc test. Scale bar: 100 μm. Data are presented as mean ± SEM, except for the Kaplan-Meier survival curve in **C**. *P < 0.05, ****P < 0.0001. #*P* < 0.05 and ##*P* < 0.01 for comparisons between hSOD1^G85R^ + vehicle and hSOD1^G85R^ + MA-5 100 nM in **B**.

To investigate the therapeutic potential of MA-5 against ALS-related neuromuscular dysfunction, we used a previously established *Drosophila* ALS model, in which the mutant human *SOD1* G85R gene is expressed in motor neurons under the control of the D42-GAL4 driver (21) (Figure 1A). This model recapitulates progressive motor decline due to the functional deterioration of motoneurons that innervate skeletal muscle. As illustrated in Figure 1A, flies were subjected to a cyclic oral administration of MA-5: filter paper soaked in 5% sucrose solution containing MA-5 was provided during the treatment period, followed by recovery on standard cornmeal food. To prevent photodegradation of the compound, flies were kept in darkness during the MA-5 treatment cycle.

Before assessing the therapeutic effect, we confirmed the biodistribution of MA-5 using BODIPY-MA-5, a fluorescently labeled derivative of MA-5. Fluorescence imaging revealed that MA-5 localized clearly to mitochondria within motor neurons of the thoracic ganglia (Supplemental Figure 1A; supplemental material available online with this article; https://doi.org/10.1172/jci.insight.200761DS1). These findings demonstrate that orally administered MA-5 can reach the mitochondria of motor neurons in the brain, indicating its ability to cross multiple biological barriers and directly act at the site of neurodegeneration to improve mitochondrial health.

To evaluate the functional consequences of MA-5 treatment, we performed a longitudinal negative geotaxis climbing assay, a well-established behavioral test of locomotor function in *Drosophila*. Climbing performance was assessed every 10 days from day 10 to day 40 in control flies, hSOD1^G85R^ flies, and hSOD1^G85R^ flies treated with MA-5 (10 or 100 nM) (Figure 1B and Supplemental Video 1). Two-way repeated-measures ANOVA revealed significant effects of treatment (*F*_3,28_ = 7.09, *P* = 0.0011) and time (*P* < 0.001), as well as a significant treatment × time interaction (*F*_9,84_ = 3.74, *P* < 0.001), indicating that the trajectory of locomotor decline differed among groups. hSOD1^G85R^ flies exhibited progressive age-dependent locomotor decline, with a marked deficit compared with control flies at day 40 (*P* < 0.001). MA-5 significantly attenuated this decline: in comparison with vehicle-treated hSOD1^G85R^ flies, 100 nM MA-5 improved climbing performance at day 30 (*P* = 0.0068) and day 40 (*P* = 0.011), whereas 10 nM MA-5 improved climbing performance at day 30 (*P* = 0.021). These results indicate that MA-5 significantly attenuates age-dependent motor decline in this *Drosophila* ALS model.

We next examined whether this preservation of motor function was accompanied by improved survival. Lifespan analysis was performed using an adult-onset *Drosophila* ALS model expressing hSOD1^G85R^ under the control of the temperature-sensitive GAL80^ts^ system. Kaplan-Meier survival analysis demonstrated that MA-5 significantly prolonged survival in comparison with vehicle-treated ALS flies (Figure 1C). Similar conclusions were obtained using 3 complementary survival tests (log rank, Wilcoxon’s, and Tarone-Ware), supporting the robustness of the survival benefit. These results indicate that MA-5 confers organismal benefits in vivo, including both preservation of locomotor function and extension of survival.

To assess whether the observed functional improvement was associated with mitochondrial integrity, we examined mitochondrial content in motor neurons of aged ALS flies by quantifying the total ATP5A-positive mitochondrial area per cell. hSOD1^G85R^ flies showed a significant reduction in total mitochondrial area per cell, and this phenotype was markedly rescued by MA-5 administration (Figure 1D), indicating preservation of mitochondrial integrity in motor neurons. Because mitochondria in neuronal processes are highly branched and frequently overlapping in confocal images, this analysis was interpreted as a quantitative measure of mitochondrial content rather than the morphology of individual mitochondria. This observation is consistent with previous studies reporting that MA-5 enhances mitochondrial structural integrity and biogenesis in disease contexts (18, 20).

We examined whether the observed functional recovery was linked to mitochondrial energy output by using the genetically encoded ATP biosensor QUEEN (22) to visualize ATP levels in motor neurons. ATP production was significantly diminished in ALS-model flies but was restored to near-normal levels following MA-5 treatment (Figure 1E), indicating a recovery of mitochondrial energy metabolism. To complement these findings, we assessed mitochondrial membrane potential using tetramethylrhodamine ethyl ester (TMRE) staining in the adult ventral nerve cord. Compared with control flies, hSOD1^G85R^ flies displayed impaired mitochondrial membrane potential, indicated by a significant reduction in TMRE fluorescence intensity (*P* < 0.0001), which was significantly restored following MA-5 administration (*P* < 0.0001) (Figure 1F). These results further support the role of MA-5 in restoring mitochondrial function at multiple levels.

Given that mitochondrial abnormalities in ALS are not restricted to neurons, we also examined skeletal muscle phenotypes. Previous studies have reported mitochondrial defects in skeletal muscle tissue of patients with ALS (15). We consistently observed a shift toward larger mitochondrial profiles in the indirect flight muscles of ALS-model flies, whereas MA-5 treatment suppressed this abnormal enlargement and restored the size distribution toward control levels (Supplemental Figure 1B).

We also performed a preliminary exploratory analysis of MA-5 in the SOD1^G93A^ mouse model. Because of the difficulty in breeding this transgenic line and maintaining high-copy animals, only one mouse per group was available for analysis, precluding statistical evaluation. Nevertheless, the MA-5–treated mouse showed qualitative improvements in locomotor performance and mitochondrial morphology compared with the control mouse, consistent with the findings obtained in our *Drosophila* experiments (Supplemental Figure 1, C–E). These observations should therefore be interpreted as preliminary supportive data, and further validation in adequately powered mammalian in vivo studies is required.

Taken together, these results demonstrate that MA-5 localizes to the mitochondria within motor neurons and restores mitochondrial content, bioenergetic output, and membrane potential in the ALS *Drosophila* model. In addition, MA-5 preserves locomotor function, and prolongs survival, supporting its therapeutic potential against ALS-associated mitochondrial dysfunction.

### MA-5 improved the impairment of mitochondrial function in ALS patient–derived fibroblasts.

**Figure 2:**
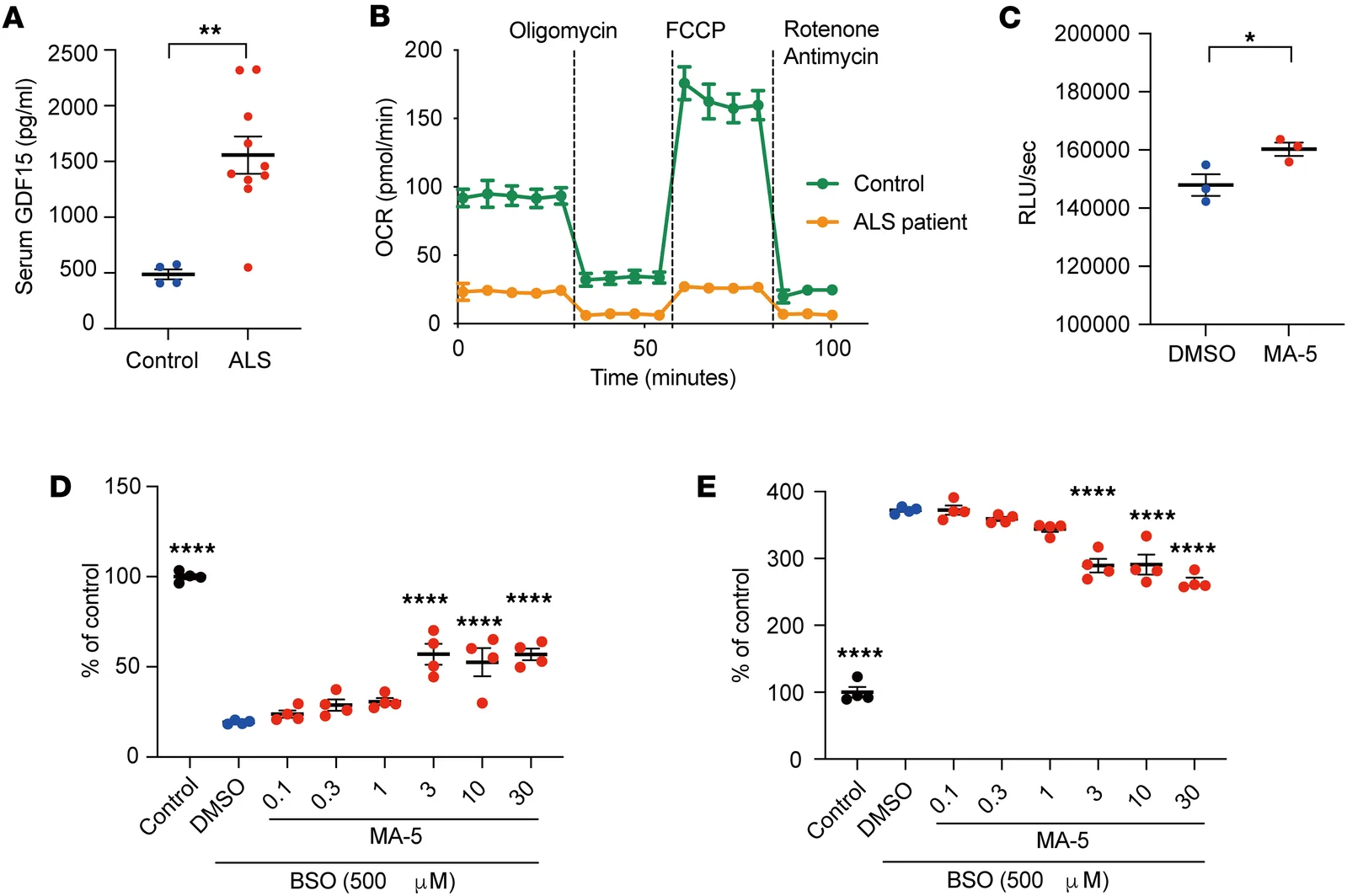
MA-5 restores mitochondrial function in fibroblasts derived from patients with ALS. (**A**) Serum GDF15 levels in patients with ALS and healthy controls (control, *n* = 4; ALS, *n* = 10). (**B**) Mitochondrial function was assessed in fibroblasts from individuals with SOD-ALS and healthy controls using a flux analyzer (*N* = 3, respectively). OCR, oxygen consumption rate. (**C**) ATP production was measured by an ATP measurement kit at 6 hours after DMSO or MA-5 treatment (10 μM) in fibroblasts from patients with SOD-ALS (*n* = 3, respectively). RLU, relative light units. (**D** and **E**) MA-5 protects against oxidative stress in fibroblasts obtained from individuals diagnosed with SOD-ALS. (**D**) Cell viability was measured by the MTT assay. (**E**) Cell damage measured by LDH assay. MA-5 was added at 0.1, 0.3, 1, 3, 10, and 30 μM (*n* = 4). Data are represented as mean ± SEM. *P < 0.05, **P < 0.01, ****P < 0.0001. **A** and **B** were analyzed by unpaired 2-tailed *t* test, while **D** and **E** were analyzed using 1-way ANOVA followed by Tukey’s multiple comparison test; each MA-5-treated group in the presence of BSO was compared with the BSO + DMSO group.

Although mitochondrial dysfunction is closely associated with ALS pathogenesis (14, 15), no biomarker for impaired mitochondria has been fully validated in patients with ALS in clinical settings. Since GDF15 has been reported as a sensitive and specific diagnostic marker for mitochondrial diseases (23, 24), we measured serum GDF15 levels in 10 patients with ALS and 4 healthy controls (patient profiles are provided in Table 1). Serum GDF15 levels were significantly higher in patients with ALS than in healthy controls (1,557.0 pg/mL vs. 487.5 pg/mL, *P* = 0.002) (Figure 2A), suggesting that mitochondrial function is impaired in patients with ALS.

Since skin fibroblasts derived from patients with ALS are known to mimic the cellular phenotypes caused by ALS (15, 25), we isolated fibroblasts from patients with *SOD1-*mutated (G85R) ALS and examined their mitochondrial function using a flux analyzer, which measures key bioenergetic parameters. This analysis revealed significant mitochondrial dysfunction in the fibroblasts derived from patients with ALS (Figure 2B). Consistent with our previous findings that MA-5 upregulates ATP production by reshaping inner mitochondrial membrane supercomplexes (18, 19), MA-5 treatment significantly increased ATP production in these fibroblasts (Figure 2C).

To further evaluate the protective effects of MA-5, fibroblasts were treated with l-buthionine (*S*,*R*)-sulfoximine (BSO), a compound that induces ROS by inhibiting glutathione synthesis and mimicking mitochondrial damage (18). Under these conditions, MA-5 significantly rescued BSO-induced cell death in ALS-derived fibroblasts in a dose-dependent manner (3–30 μM) (Figure 2D). This protective effect of MA-5 was further confirmed by a reduction in lactate dehydrogenase (LDH) levels under mitochondrial stress (3–30 μM) (Figure 2E).

To validate the effectiveness of MA-5 in other types of ALS, we isolated fibroblasts from 2 patients with sporadic ALS without known ALS gene mutations. One of the fibroblasts from patients with sporadic ALS exhibited reduced mitochondrial function, similar to that of fibroblasts from patients with mutated *SOD1* (G85R) ALS (Supplemental Figure 2A). MA-5 treatment increased ATP production in both fibroblast samples from patients with sporadic ALS (Supplemental Figure 2B). To further confirm the effect of MA-5 on mitochondrial ATP production, we analyzed isolated mitochondria. MA-5 treatment increased ATP production in isolated mitochondria (Supplemental Figure 2C). BSO-induced cell death and LDH release were attenuated by MA-5 in sporadic ALS–derived fibroblasts in a dose-dependent manner up to 10 μM, whereas 30 μM MA-5 appeared to be less effective or potentially harmful (Supplemental Figure 2, D and E).

These findings indicate that MA-5 ameliorates mitochondrial dysfunction in fibroblasts derived from patients with various types of ALS, including those with *SOD1* mutations and sporadic ALS, highlighting its potential as a therapeutic intervention for ALS.

### MA-5 restored the phenotypes of iPSC-derived motor neurons from patients with ALS.

**Figure 3:**
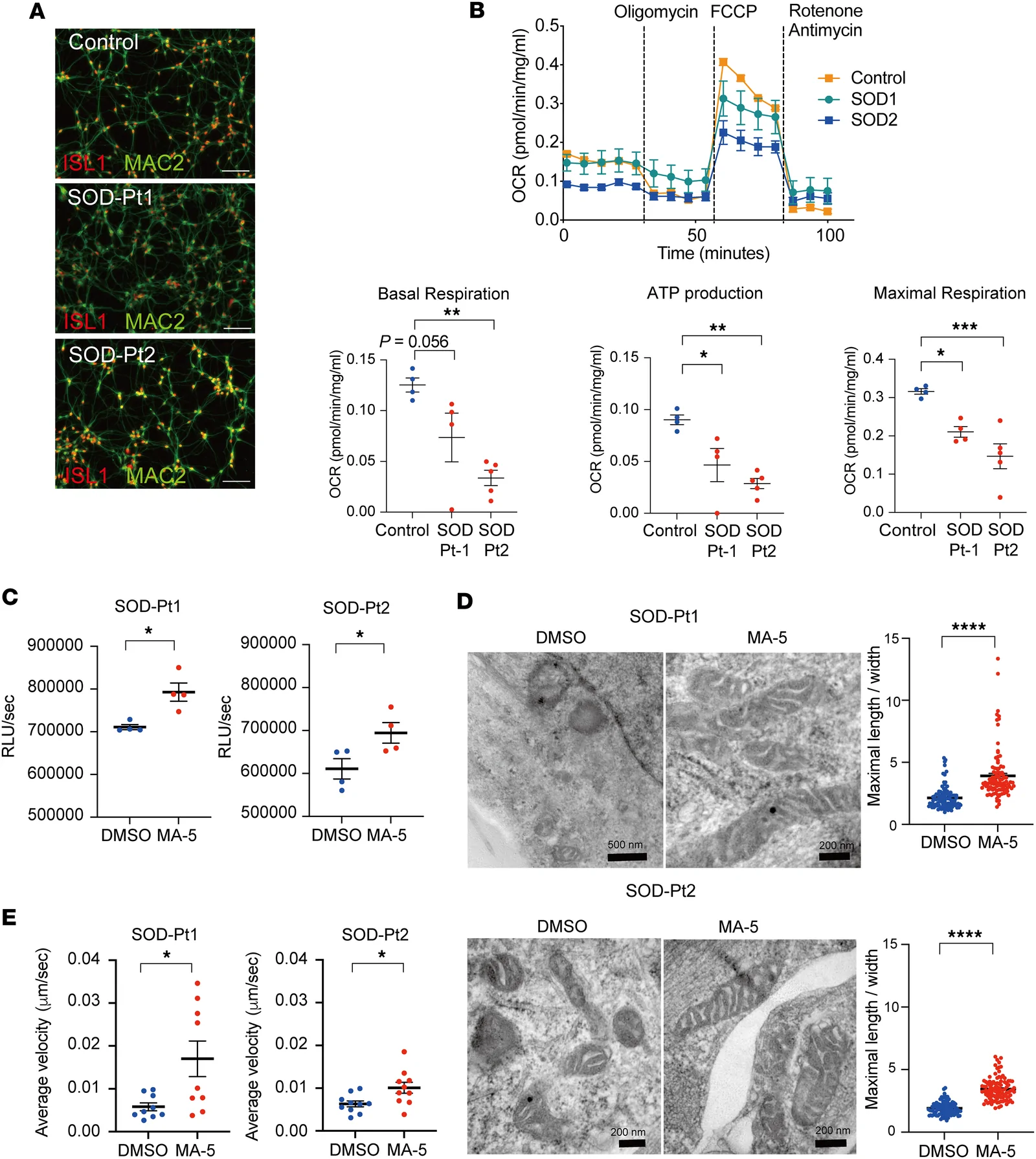
MA-5 alleviates mitochondrial dysfunction and structural abnormalities in motor neurons derived from *SOD1*-ALS-iPSCs. (**A**) Motor neurons differentiated from *SOD1*-ALS-iPSCs (2 patients; SOD-Pt1 and SOD-Pt2). Scale bars: 100 μm. Motor neuron markers: ISL1 (ISL LIM homeobox 1) and MAC2 (galectin 3). (**B**) A flux analyzer was used to assess mitochondrial function in motor neurons derived from *SOD1*-ALS-iPSCs and healthy controls (control, *n* = 4; SOD1, *n* = 4; SOD2, *n* = 5). Oxygen consumption rate (OCR) values were normalized to the protein concentration of the motor neurons. (**C**) ATP production at 6 hours after DMSO or MA-5 treatment (100 nM) in motor neurons from *SOD1*-ALS-iPSCs (*n* = 4, respectively). RLU, relative light units. (**D**) Mitochondrial malformation measured as the ratio of maximal length to width (100 mitochondria measured) in motor neurons from *SOD1*-ALS-iPSCs with or without MA-5 treatment (100 nM). (**E**) Mitochondrial movement velocity in motor neurons from *SOD1*-ALS-iPSCs (SOD-Pt1, *n* = 9; SOD-Pt2, *n* = 10) with or without MA-5 treatment (100 nM). Data are represented as mean ± SEM. *P < 0.05, **P < 0.01, ***P < 0.001, ****P < 0.0001. **B** was analyzed by 1-way ANOVA, while **C**–**E** were analyzed using unpaired 2-tailed *t* tests.

In addition to the effects of MA-5 on skin fibroblasts derived from patients with ALS, we generated iPSCs from patients with ALS carrying *SOD1* mutations (*SOD1*-ALS-iPSCs; SOD-Pt1 and SOD-Pt2 from two ALS patients, respectively) and differentiated these iPSCs into motor neurons, as previously described, given that motor neurons are not easily procured from patients with ALS (26) (Figure 3A). Motor neurons derived from *SOD1*-ALS-iPSCs showed impaired mitochondrial respiratory parameters compared with those derived from healthy control iPSCs, although the extent of impairment varied between patient-derived lines (Figure 3B). Basal respiration was significantly reduced in one of the two ALS-derived motor neuron lines, whereas ATP production and maximal respiration were consistently impaired in both lines (Figure 3B). Consistent with the results observed in fibroblasts derived from patients with ALS, ATP production was significantly increased by MA-5 treatment in *SOD1*-ALS-iPSC–derived motor neurons (Figure 3C). We also examined multiple concentrations of MA-5 to determine the optimal dose for enhancing ATP production. These analyses suggested that 100 nM MA-5 effectively increased ATP production, whereas concentrations of 10 μM or higher appeared to be ineffective or potentially harmful in this experimental setting. Electron microscopy further revealed mitochondrial malformations in *SOD1*-ALS-iPSC–derived motor neurons, which were rescued by MA-5 treatment (Figure 3D). Mitochondrial dysfunction affects both mitochondrial dynamics and morphology (27). To investigate this, we compared the mitochondrial dynamics in *SOD1*-ALS-iPSC–derived motor neurons treated with either dimethylsulfoxide (DMSO) or MA-5. The average velocity of mitochondrial movement was significantly improved by MA-5 treatment (0.005 vs. 0.017 μm/s and 0.006 vs. 0.010 μm/s, *P* = 0.017 and *P* = 0.019, respectively) (Figure 3E and Supplemental Video 2). Similar results were observed in motor neurons derived from iPSCs of patients with ALS carrying *FUS^H517D/H517D^* gene mutations (*FUS*-ALS-iPSC–derived motor neurons) (28). Mitochondrial function parameters, including basal respiration, ATP production, and maximal respiration, were significantly lower in *FUS*-ALS-iPSC–derived motor neurons than in those derived from healthy controls (Supplemental Figure 3A). MA-5 treatment significantly improved ATP production, mitochondrial morphology, and mitochondrial dynamics, consistent with the results observed in *SOD1*-ALS-iPSC–derived motor neurons (Supplemental Figure 3, B–D, and Supplemental Video 3). These findings indicate that MA-5 improves mitochondrial function in motor neurons derived from patients with ALS and suggest therapeutic potential for treating ALS phenotypes.

### MA-5 likely treated iPSC-derived motor neurons by manipulating the expression of ALS-related genes and mitochondrial genes.

**Figure 4:**
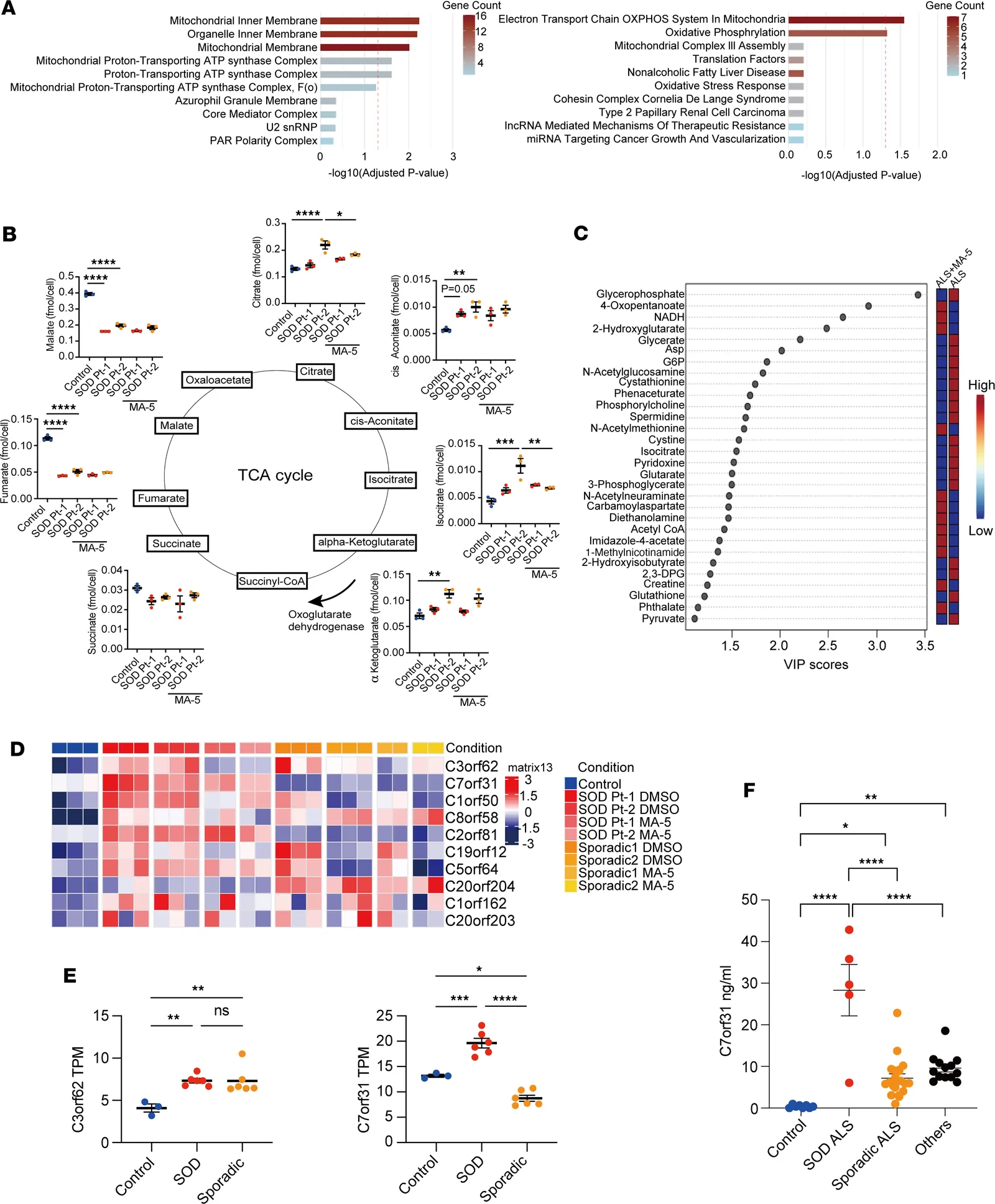
Effect of MA-5 on iPSC-derived motor neurons and potential clinical markers identified in ALS-iPSCs. (**A**) Enrichment analyses of gene expression changes caused by MA-5 treatment (100 nM) in motor neurons derived from *SOD1*-ALS-iPSCs. The enrichment analysis was performed using Gene Ontology Cellular Component 2023 (left) and WikiPathways 2023 (right), which are integrated in the Enrichr tool (https://maayanlab.cloud/Enrichr/). Mutual genes altered by MA-5 in SOD-Pt1 and SOD-Pt2 lines were analyzed. (**B**) Metabolites involved in the tricarboxylic acid (TCA) cycle in motor neurons derived from healthy controls and *SOD1*-ALS-iPSCs with or without MA-5 (10 μM) (*N* = 3). (**C**) Variable importance in projection (VIP) scores in motor neurons derived from *SOD1*-ALS-iPSCs with or without MA-5 (10 μM) (*N* = 6, respectively). (**D**) The top 10 ORF genes increased in motor neurons derived from *SOD1*-ALS-iPSCs and decreased by MA-5 (100 nM). Gene expression in motor neurons derived from sporadic-ALS-iPSCs with or without MA-5 (100 nM) is also shown. (**E**) C3orf62 and C7orf31 expression in motor neurons from *SOD1*-ALS-iPSCs, sporadic-ALS-iPSCs, and healthy controls (control, *n* = 3; SOD, *n* = 6; sporadic, *n* = 6). TPM, transcripts per kilobase million. (**F**) Plasma C7orf31 levels in healthy controls (*n* = 8), patients with SOD-ALS (*n* = 5), patients with sporadic ALS (*n* = 21), and those with other neurological diseases (*n* = 13; neuropathy with liability to pressure palsies, dermatomyositis, Parkinson’s disease, and spinocerebellar degeneration). Data are represented as mean ± SEM. *P < 0.05, **P < 0.01, ***P < 0.001, ****P < 0.0001. **B**, **E**, and **F** were analyzed using 1-way ANOVA.

We next investigated transcriptomic alterations in *SOD1*-ALS-iPSC–derived motor neurons to elucidate the molecular mechanisms by which MA-5 ameliorates disease-associated phenotypes. Differential gene expression analysis was performed by comparison of *SOD1*-ALS-iPSC–derived motor neurons treated with or without MA-5. Genes commonly regulated by MA-5 in both SOD-Pt1 and SOD-Pt2 lines were subjected to gene set enrichment analysis. The analysis revealed significant enrichment of mitochondria-related gene sets, including those associated with the mitochondrial inner membrane and the oxidative phosphorylation (OXPHOS) system (Figure 4A). These findings suggest that MA-5 exerts its therapeutic effects, at least in part, by modulating mitochondrial gene expression and restoring mitochondrial function in ALS motor neurons.

We further analyzed the metabolites in *SOD1*-ALS-iPSC–derived motor neurons using metabolomics, which enabled the comprehensive analysis of over 600 metabolites. The results indicated that metabolites upstream of succinate in the tricarboxylic acid (TCA) cycle tended to accumulate in *SOD1*-ALS-iPSC–derived motor neurons compared with those in healthy controls, whereas metabolites downstream of succinate were significantly decreased (Figure 4B). Reduced activity of oxoglutarate dehydrogenase, an enzyme that converts α-ketoglutarate to succinyl-CoA, has been reported in the spinal cords of hSOD1^G93A^ mice, a known ALS model carrying mutated human *SOD1* (29, 30). We also confirmed that similar metabolic changes were observed in the cerebrospinal fluid of patients with ALS, where metabolites upstream of succinate were significantly increased compared with those in disease controls (patients with spinocerebellar degeneration) (Supplemental Figure 4A). These metabolic findings suggest that the phenotypes observed in *SOD1*-ALS-iPSC–derived motor neurons resemble those observed in other ALS models, specifically regarding disrupted metabolic pathways. However, MA-5 did not reverse the metabolic changes in the TCA cycle observed in ALS motor neurons, suggesting that its mechanism of action may involve pathways independent of the TCA cycle.

To identify additional metabolites affected by MA-5 treatment, we performed comparative metabolomic profiling of *SOD1*-ALS-iPSC–derived motor neurons treated with or without MA-5, using variable importance in projection (VIP) scores (Figure 4C). Among the top-ranked metabolites, a reduction in glycerophosphate and an elevation in NADH levels were particularly notable following MA-5 treatment. These changes suggest a potential downregulation of the glycerophosphate shuttle pathway. The glycerophosphate shuttle pathway is known to contribute to mitochondrial ROS production, which plays a pathogenic role in neurodegenerative diseases, including ALS (31–33). We further examined whether pharmacological inhibition of the glycerophosphate shuttle reduces mitochondrial ROS production in *SOD1*-ALS-iPSC–derived motor neurons using iGP-1, a specific inhibitor of mitochondrial glycerol-3-phosphate dehydrogenase (mGPDH), a key enzyme in this pathway (34). iGP-1 treatment significantly reduced mitochondrial ROS production in these cells (Supplemental Figure 4B). These findings imply that MA-5 may attenuate dysregulation of the glycerophosphate shuttle, thereby partially restoring mitochondrial redox balance and function.

### A potential biomarker to diagnose subtypes of ALS and monitor the effect of MA-5 treatment.

Based on the established in vitro disease model using ALS-iPSC–derived motor neurons, we further investigated potential clinical markers to monitor the effects of MA-5 treatment. Since ORF genes have been reported as novel clinical markers (35), we focused on ORF genes. RNA-seq analysis revealed that *C3orf62* and *C7orf31* were upregulated in *SOD1*-ALS-iPSC–derived motor neurons, following downregulation by MA-5 treatment, suggesting that these genes may serve as markers for monitoring the therapeutic effects of MA-5 (Figure 4D).

Notably, while *C3orf62* was also upregulated in sporadic-ALS-iPSC–derived motor neurons, *C7orf31* expression did not increase in these cells (Figure 4, D and E). These findings suggest that *C7orf31* may serve as a potential clinical marker for ALS subtyping, aiding in the distinction between *SOD1*-mutated and sporadic forms of the disease.

To validate these findings, we measured the concentration of C7orf31 protein in the plasma of patients with *SOD1*-ALS and those with sporadic ALS. The plasma concentration of C7orf31 protein was significantly higher in patients with *SOD1*-ALS than in those with sporadic ALS (28.32 ± 12.37 ng/mL vs. 7.20 ± 4.52 ng/mL, *P* < 0.0001) (Figure 4F). This result indicates that plasma C7orf31 may be a promising biomarker for diagnosing ALS with *SOD1* mutations and for monitoring the effect of MA-5 therapy.

## Discussion

This study demonstrated that MA-5, a novel compound that targets mitochondrial dysfunction, significantly improved ALS phenotypes in *Drosophila* models, patient-derived fibroblasts, and iPSC-derived motor neurons from individuals with ALS. Notably, this work represents one of the few studies to systematically integrate in vivo, ex vivo, and human stem cell–derived systems, establishing a comprehensive validation framework for a candidate therapeutic. While our investigation primarily focused on ALS caused by *SOD1* mutations, MA-5 was also effective against other forms of ALS, including *FUS*-mutated and sporadic ALS. MA-5 enhanced mitochondrial ATP production and improved ALS phenotypes in both *Drosophila* and patient-derived cells.

Approximately 90% of ATP production in neurons is generated via mitochondrial oxidative phosphorylation (36). However, mitochondrial transport and morphology become abnormal in the early stages of ALS (37, 38). Although increased ATP production can protect neurons, forcing the impaired mitochondria to overwork may simultaneously induce oxidative stress (39). In contrast, MA-5 enhances mitochondrial ATP production without inducing oxidative stress, acting through a distinct mechanism that involves promoting ATP synthase oligomerization and respiratory supercomplex assembly via its interaction with mitofilin (18). This unique mode of action positions MA-5 as a promising therapeutic candidate for neurodegenerative disorders characterized by disrupted mitochondrial homeostasis.

Targeting mitofilin, a core component of the mitochondrial contact site and cristae organizing system (MICOS) complex, may be a critical approach in the treatment of ALS. For instance, in CHCHD10-related ALS, mutations in *CHCHD10* disrupt the MICOS complex, resulting in mitochondrial dysfunction, loss of crista junctions, and impaired oxidative phosphorylation. This occurs because CHCHD10 interacts with mitofilin, a protein crucial for mitochondrial integrity, within the MICOS complex (40, 41). Furthermore, in ALS associated with *C9orf72* G4C2 repeat expansions, *Drosophila* models expressing poly(GR) peptides, which are toxic dipeptide repeat proteins, exhibit altered MICOS dynamics and disrupted intra-subunit interactions, potentially contributing to mitochondrial dysfunction in patients with ALS. This implicates MICOS as a key player in the pathogenesis of *C9orf72*-related ALS (42). These findings suggest that the MICOS complex, particularly mitofilin, is a promising therapeutic target for ALS, highlighting the relevance of MA-5’s pharmacological mechanism.

To gain further insight into the molecular mechanisms by which MA-5 affects mitochondrial pathways, we performed a focused analysis of MitoCarta3.0-annotated mitochondrial genes within our RNA-seq dataset. Using transcriptome-wide edgeR generalized linear model (GLM) analysis, which included 46,287 genes, we identified 82 mitochondrial genes that were concordantly upregulated by MA-5 in both independent SOD1 patient-derived motor neuron lines, HPS0476 and HPS0485. Among these genes, four — *COX8A*, *COX7A2*, *NDUFAB1*, and *TMEM126A* — showed statistically significant upregulation in both lines. The upregulation of these genes following MA-5 treatment may reflect an adaptive mitochondrial-nuclear response rather than a direct transcriptional effect of MA-5. MA-5 has been reported to bind mitofilin/MIC60 and improve mitochondrial crista organization, ATP synthase oligomerization, and respiratory chain function. In this context, improved mitochondrial bioenergetics may trigger adaptive mitochondria-to-nucleus signaling, potentially involving PGC-1/NRF–associated mitochondrial biogenesis programs, leading to coordinated upregulation of nuclear-encoded oxidative phosphorylation–related genes. Such transcriptional changes may support the assembly, stabilization, or functional restoration of respiratory chain complexes, particularly complexes I and IV. Consistent with this interpretation, COX8A and COX7A2 encode nuclear-encoded subunits of cytochrome *c* oxidase, whereas NDUFAB1 and TMEM126A are associated with complex I function and assembly. Thus, their induction is consistent with the restoration of mitochondrial respiratory capacity and mitochondrial homeostasis in MA-5–treated iPSC-derived motor neurons.

In this study, we identified *C3orf62* and *C7orf31* as potential diagnostic markers for *SOD1*-mutated ALS and as markers for monitoring the therapeutic effects of MA-5. Notably, *C7orf31* may also serve as a marker for distinguishing *SOD1*-mutated ALS from sporadic ALS. Elevated *C7orf31* has been reported in the postmortem spinal cord of patients with ALS, which might corroborate this finding (43). Since the median survival time from ALS onset is short, early diagnosis and rapid monitoring of disease progression and treatment responses are critical. While the clinical course and phenotypes of ALS vary depending on the causal genes (3), genetic testing is labor-intensive, costly, and time-consuming. Therefore, useful clinical biomarkers of ALS are urgently required. Although studies have shown that creatine kinase levels are elevated in patients with ALS, the underlying mechanism remains unclear (44). Despite decades of research, no novel clinical markers of ALS have been developed so far. This study paves the way for the discovery of new ALS biomarkers using patient-derived iPSC models.

This study has several limitations. While MA-5 was shown to be effective in SOD1-mutated, FUS-mutated, and sporadic ALS, the study primarily focuses on these forms and does not include other ALS subtypes, which may limit the broader applicability of the findings. Additionally, the study relies heavily on *Drosophila*, patient-derived fibroblasts, and ALS patient iPSC–derived motor neurons, which, although valuable, may not fully recapitulate the complexities of ALS pathology in humans. The number of ALS patient–derived cell lines, particularly iPSC-derived motor neuron lines, was also limited. Further validation in more comprehensive in vivo models, including adequately powered mammalian models, and ultimately in human clinical trials, will be required to confirm the therapeutic potential of MA-5. The identification of C3orf62 and C7orf31 as potential biomarkers requires further validation in larger ALS cohorts. In our preliminary analysis of patients with *SOD1*-ALS, elevated plasma C7orf31 levels were observed in some patients with severe clinical phenotypes. Although this finding raises the possibility that plasma C7orf31 may be related to disease severity, the small sample size precludes any conclusion regarding a quantitative correlation. Further clinical studies in larger and longitudinal cohorts are required to confirm the utility of C7orf31 as a biomarker. Future studies should assess the specificity and sensitivity of these markers across ALS subtypes, including in presymptomatic patients, to establish their clinical utility in diagnosing and monitoring disease progression.

In conclusion, this study demonstrated that MA-5, a novel mitochondria-targeting compound, is a promising candidate for ALS treatment. Given that MA-5 has completed phase I clinical trials, its feasibility as a new therapy for ALS appears to be high. Additionally, we identified new clinical markers that can monitor the effectiveness of MA-5 and distinguish between ALS subtypes. Overall, our findings contribute to the development of novel clinical strategies for the treatment and diagnosis of ALS.

## Methods

Additional reagents and resources are listed in Table 2.

### Sex as a biological variable.

Human clinical samples and cell lines were derived from both female and male. ALS model mice also included male (SOD1-Ctrl) and female (SOD1-MA-5). In the *Drosophila* experiments, male flies were used for the climbing assay and all imaging analyses, whereas the lifespan analysis included both males and females, whose survival data were pooled. Males were used elsewhere to match the original characterization of this human SOD1 (hSOD1) model, in which the behavioral and histological analyses were performed on males (21), and to remove sex as a source of variability.

### Human participants.

To investigate the level of mitochondrial dysfunction in patients with ALS, serum levels of GDF15 were measured in patients with ALS and healthy controls (Figure 2A). Human skin fibroblasts were isolated from patients with ALS and healthy controls as previously described to examine the mitochondrial function in cells from patients with ALS (18) (Figure 2, B–E). The patient profiles are presented in Tables 1 and 3. iPSCs derived from patients with ALS carrying the SOD mutation, HPS0476 and HPS0485, were obtained from the RIKEN Cell Bank in Japan. iPSCs derived from patients with sporadic ALS were generated in our laboratory. In contrast, iPSCs derived from patients with ALS carrying the FUS gene mutation were described previously (28).

### Drosophila stocks and MA-5 treatment.

The GAL4-UAS expression system was used to direct the transgene expression in specific cell types. The D42-GAL4 driver line was used for motor neuron–specific expression, whereas the *w^1118^* strain, a commonly used genetic background for transgenic *Drosophila*, served as the control. The following stocks were obtained from the Bloomington *Drosophila* Stock Center (Indiana University, Bloomington, Indiana, USA): *w^1118^* (stock 3605), *D42-GAL4* (stock 8816), *UAS-hSOD1^G85R^* (stock 33608), *UAS-mCD8:GFP* (stock 5137), *elav-GAL4^c155^* (stock 458), and *tubP-GAL80^ts^* (stock 7019). The *elav-GAL4^c155^; tub-GAL80^ts^/CyO* stock used for the lifespan assay was generated in this laboratory by combining of the latter two. Flies were maintained at 25°C under standard conditions.

MA-5 was dissolved in 5% sucrose solution and administered orally via filter paper placed in vials. Flies were treated with MA-5 (10 or 100 nM) for 3 days, followed by 2 days on standard food. To minimize MA-5 photodegradation, flies were kept in darkness during drug treatment.

### Drosophila climbing assay.

Flies were collected within 4 days of eclosion and divided into independent vials containing 15–20 flies each. Locomotor function was assessed every 10 days from day 10 to day 40 using a negative geotaxis assay. For each trial, flies were gently tapped to the bottom of an empty vial and allowed to climb for 20 seconds. The climbing pass rate was calculated as the percentage of flies that reached or exceeded a height of 5 cm within 20 seconds. Each vial was tested 3 times per session with approximately 1-minute intervals between trials, and the mean of the 3 trials was used for subsequent analysis. Each vial was treated as 1 biological replicate. For each treatment group, a total of 11 vials were analyzed; 8 vials were tracked at all time points from day 10 to day 40, whereas 3 additional vials were included from day 30 onward. Consequently, the number of vials per group was 8 at days 10 and 20 and 11 at days 30 and 40.

### Immunohistochemistry and image analysis in Drosophila.

To visualize mitochondria, samples were stained with an anti-ATP5A antibody (1:500; Abcam, 14748). Neuronal nuclei were labeled using Rat-Elav-7E8A10 antibody (1:500; Developmental Studies Hybridoma Bank, 7E8A10), and muscle actin was visualized using Alexa Fluor 633 phalloidin. Samples were imaged using a Zeiss LSM 980 confocal microscope. Total ATP5A-positive mitochondrial area was quantified using Fiji/ImageJ (NIH). Biodistribution studies were performed using BODIPY-MA-5.

### TMRE staining and quantification.

For TMRE staining, third-instar larval ventral nerve cords (thoracic ganglia) were dissected in HL3.1 buffer and incubated with 100 nM TMRE in HL3.1 buffer for 12 minutes at 25°C. After staining, samples were washed twice (30 seconds each) in HL3.1 buffer containing 25 nM TMRE. Live imaging was performed on freshly prepared samples, with all acquisitions completed within 1 hour of dissection. Images were acquired on a Zeiss LSM980 confocal microscope with a ×20 objective (TMRE excitation ~555 nm). TMRE fluorescence intensity was quantified in Fiji from a single region of interest spanning the T1–T2 segments of each sample, and normalized to the mean of the control group within the same experimental batch to account for inter-batch variability.

### Generation of transgenic QUEEN flies and QUEEN imaging and analysis.

The UAS-QUEEN construct was generated from pN1-QUEEN-7μ (Addgene, 129307) and inserted into the attP40 landing site by WellGenetics. Intracellular ATP levels were measured using the genetically encoded ATP biosensor QUEEN. Thoracic ganglia were dissected in HL3.1 buffer and imaged within 1 hour using a Zeiss LSM980 confocal microscope (45). Fluorescence images were acquired at 405 nm and 488 nm excitation wavelengths, and ATP levels were estimated from the 405/488 fluorescence ratio (22). Three control flies, 4 vehicle-treated hSOD1^G85R^ flies, and 4 MA-5–treated hSOD1^G85R^ flies were analyzed. For each fly, regions within the T1–T2 segments of the thoracic ganglion with low background fluorescence and clear QUEEN signal were selected as regions of interest. A total of 1,144 cells from control flies, 551 cells from vehicle-treated hSOD1^G85R^ flies, and 549 cells from MA-5–treated hSOD1^G85R^ flies were quantified.

### Drosophila lifespan assay.

Adult-onset neuronal expression of hSOD1^G85R^ was induced using the *elav-GAL4^c155^ tub-GAL80^ts^* driver system. *UAS-hSOD1^G85R^* flies, carrying transgene insertions on the second and third chromosomes, were introgressed into a *w^1118^* background before use. Both male and female flies were used. Adult-onset ALS-model flies were *elav-GAL4^c155^/+* (females) or *elav-GAL4^c155^/Y* (males)*;* *tub-GAL80^ts^/UAS-hSOD1^G85R^; UAS-hSOD1^G85R^/+*, and the corresponding driver-only genotype, *elav-GAL4^c155^/+* or */Y; tub-GAL80^ts^/+*, was used as the control.

Flies were maintained at 18°C during development to suppress GAL4 activity. Newly eclosed adults were collected on day 1 after eclosion, distributed across 3 independent vials per condition (control, 194 flies in 11 vials; hSOD1^G85R^ + vehicle, 110 in 7 vials; + MA-5 10 nM, 121 in 7 vials; + MA-5 100 nM, 122 in 7 vials, with 11–22 flies per vial), and maintained at 18°C. On day 4 after eclosion, flies were shifted to 29°C to induce pan-neuronal hSOD1^G85R^ expression; this day was designated day 0 post-induction. MA-5 was administered at 10 nM and 100 nM in 5% sucrose containing 0.01% DMSO via filter paper placed in empty vials, and vehicle-treated flies received 5% sucrose containing 0.01% DMSO. Flies were alternately transferred between drug-treatment vials and standard food vials every other day without anesthesia. Survival was monitored daily until day 50 post-induction.

### SOD1^G93A^ mouse model studies.

All animal procedures were performed in accordance with protocols approved by the Animal Experiment Committee of the National Institute of Neuroscience, National Center of Neurology and Psychiatry. SOD1^G93A^ transgenic mice, which express a G93A mutant form of human SOD1, were obtained from The Jackson Laboratory (002726). Heterozygous (SOD1^G93A^) males were bred with wild-type female C57BL/6J mice (Japan SLC) as previously reported (46). SOD1^G93A^ mice were fed standard chow with or without MA-5 (0.02%) starting at 40 days of age. Rotarod and grip strength tests were performed at 100 days of age. Mice were sacrificed at 120 days of age, and motor neurons in the lumbar spinal cord were analyzed by electron microscopy.

### Measurement of serum GDF15 levels.

Blood samples were collected from patients with ALS visiting Tohoku University Hospital and stored at –80°C until analysis. Serum GDF15 levels were measured in patients with ALS and healthy controls using the Human GDF-15 ELISA Kit (R&D Systems).

### Isolation of human skin fibroblasts and assays.

Human skin fibroblasts were isolated from patients with ALS and healthy controls as previously described (18). The cells were cultured in low-glucose DMEM (1.0 g/L) supplemented with 10% fetal bovine serum. Cell viability was assessed using Cell Count Reagent SF (Nacalai Tesque) 72 hours after treatment with BSO (Wako Pure Chemical Industries) or DMSO. Lactate dehydrogenase (LDH) levels were measured using the LDH Cytotoxicity Detection Kit (Takara). ATP production was evaluated using an ATP measurement kit (Toyo Ink) 6 hours after DMSO or MA-5 treatment (10 μM). Mitochondria were isolated using the Mitochondria Isolation Kit for Cultured Cells according to the manufacturer’s instructions (Thermo Fisher Scientific, 89874). The patient profiles are provided in Table 3.

### Mitochondrial function measurement.

Oxygen consumption in fibroblasts and iPSC-derived motor neurons was measured using the Flux Analyzer XF24 or XF96 (Agilent), as previously described (18). Cells were incubated in the assay medium without CO_2_ for 60 minutes before sequential injections of mitochondrial oxidative phosphorylation inhibitors. Because iPSC-derived motor neurons can show variability in seeding density and attachment efficiency, the data were normalized to protein concentration.

### Culture and differentiation of iPSCs.

iPSCs derived from patients with ALS carrying the SOD mutation, HPS0476 and HPS0485, were obtained from the RIKEN Cell Bank in Japan and maintained as described previously (47). iPSCs derived from patients with sporadic ALS were generated in our laboratory using the Epi5 Episomal iPSC Reprogramming Kit (Life Technologies). Motor neurons were differentiated using a previous protocol (26). Briefly, iPSCs were dissociated and seeded on Matrigel-coated plates. On the following day, the iPSC medium was replaced with a neuron differentiation medium (1:1 mixture of DMEM/F12 and Neurobasal medium [Thermo Fisher Scientific] with 0.5× N2 and B27, 0.1 mM ascorbic acid [Sigma Aldrich], and 1× GlutaMAX [Life Technologies]) supplemented with 3 μM CHIR99021 (Cayman), 2 μM DMH1 (Cayman), and 2 μM SB431542 (Cayman) to differentiate iPSCs into neuron epithelial progenitors (NEPs). On day 6, the cells were dissociated and split at 1:6 with the neuron differentiation medium, including 1 μM CHIR99021, 2 μM DMH1, 2 μM SB431542, 0.1 μM retinoic acid (Cayman), and 0.5 μM purmorphamine (Cayman). The cells were maintained under these conditions for 6 days so that the NEPs could differentiate into OLIG2^+^ motor neuron progenitors (MNPs). To produce motor neurons, OLIG2^+^ MNPs were dissociated with dispase and cultured in the differentiation medium with 0.5 μM retinoic acid and 0.1 μM purmorphamine. Under these conditions for 6 days, the motor neurons obtained were dissociated and plated on Matrigel-coated plates in the differentiation medium, including 0.5 μM retinoic acid, 0.1 μM purmorphamine, and 0.1 μM compound E (Calbiochem) for maturation. In contrast, iPSCs derived from patients with ALS carrying the FUS gene mutation were described previously (28) and differentiated as previously described (28). For experiments examining inhibition of the glycerophosphate shuttle, iPSCs were differentiated into motor neurons using a previously reported protocol (48).

### Immunofluorescence.

Immunofluorescence assays were performed as previously described (47). iPSC-derived motor neurons were fixed in 4% paraformaldehyde and stained with primary and secondary antibodies (Table 2).

### Mitochondrial motility analysis.

Live-cell imaging of the motor neurons stained with MitoTracker Green was performed using a KEYENCE BZ-X700 microscope. Time-lapse videos were analyzed for cell migration using the VW-H2MA software (KEYENCE) (20).

### Electron microscopy analysis.

Mitochondrial dimensions in iPSC-derived motor neurons were measured as previously reported (18). For *Drosophila*, the electron microscopy images were analyzed using size-based categorization (49).

### RNA sequencing analysis.

RNA sequence libraries were prepared using QIAseq Stranded RNA Library Kits (QIAGEN, catalog 180451) following the manufacturer’s instructions. The Illumina libraries were converted into circular single-stranded DNA libraries using the MGI Easy Universal Library Conversion Kit (App-A, MGI Tech). The converted libraries were sequenced on a DNBSEQ-G400RS platform (MGI Tech) using the 2 × 150 bp paired-end mode. RNA sequencing (RNA-seq) was performed using DNAFORM (Yokohama). Sequence reads in FASTQ format were evaluated for quality using FastQC (https://www.bioinformatics.babraham.ac.uk/projects/fastqc/), and low-quality reads were removed using Trimmomatic-0.39 (https://github.com/usadellab/Trimmomatic). High-quality reads were mapped to the GRCh38 reference genome using STAR (v2.7.10a; https://github.com/alexdobin/STAR), a splice-aware aligner. Gene- and transcript-level abundances were quantified using StringTie (v2.2.0; https://ccb.jhu.edu/software/stringtie/), which provides accurate expression estimates. Gene expression levels were analyzed using the edgeR package (v3.15) in Bioconductor, which normalizes the data and identifies differentially expressed genes. Functional enrichment analyses were performed using EnrichR, focusing on the mutual genes affected by MA-5 treatment in SOD-Pt1 and SOD-Pt2 cell lines to identify relevant pathways and biological processes.

### Metabolomics analysis.

Metabolomics analysis was performed as previously described (50, 51). Metabolites were initially separated by capillary electrophoresis based on their charge and size. This method enables high-resolution separation of small molecules under an electric field. Metabolites were then selectively detected using mass spectrometry, which monitors a broad range of mass-to-charge (*m*/*z*) values to capture a comprehensive metabolite profile. Variable importance in projection (VIP) scores were calculated and analyzed using MetaboAnalyst (https://www.metaboanalyst.ca/) to identify the metabolites that contributed most significantly to the group differences between patients with ALS and healthy controls.

### Glycerophosphate shuttle inhibition.

Differentiated motor neurons derived from HPS0485 iPSCs were seeded in 96-well plates and incubated with or without iGP-1 (10 μM) for 1 hour. MitoSOX Red mitochondrial superoxide indicator (Invitrogen, M36008; 0.5 μM) was then added to quantify mitochondrial ROS production. Six images were acquired from each well using a BZ-X810 fluorescence microscope (KEYENCE). MitoSOX fluorescence intensity was normalized to cell number, which was determined by Hoechst staining. The average MitoSOX fluorescence intensity per cell in each well was used as the final value for analysis.

### C7orf31 measurement.

Plasma C7orf31 protein levels were measured using a C7orf31 ELISA Kit (MyBioSource, catalog MB9340367).

### Statistics.

Statistical analyses were performed using GraphPad Prism software. For comparisons between 2 groups, unpaired 2-tailed *t* tests were used, while 1-way analysis of variance (ANOVA) was applied for multiple comparisons, followed by Tukey’s correction. For the *Drosophila* climbing assay, each vial was treated as 1 biological replicate. Differences in climbing performance over time were analyzed using 2-way repeated-measures ANOVA, with treatment group as the between-subjects factor and day as the within-subject factor, followed by Bonferroni correction for multiple comparisons. For *Drosophila* lifespan analysis, survival data were analyzed using Kaplan-Meier estimation. Pairwise comparisons were performed using log-rank (Mantel-Cox), Wilcoxon’s (Breslow), and Tarone-Ware tests. Kaplan-Meier estimation and survival statistics were performed using custom Python 3 scripts. Statistical significance was set at a *P* value of <0.05.

### Study approval.

All these studies were approved by the Ethics Committee of the Medical School of Tohoku University (2018-2-254, 2021-1-905, 2021-1-901, and 2022-1-823). Ethical guidelines were strictly followed to ensure the integrity and confidentiality of the patient samples.

### Data availability.

All unique/stable materials generated in this study are available from Takafumi Toyohara with a completed material transfer agreement. The raw RNA sequencing data generated in this study from motor neurons derived from ALS-iPSCs were deposited in the NCBI’s Sequence Read Archive under BioProject accession number PRJNA1272663 (https://www.ncbi.nlm.nih.gov/bioproject/?term=PRJNA1272663). The numerical source data underlying the figures are provided in the accompanying Supporting Data Values file. Requests for further information or for resources and reagents should be directed to and will be fulfilled by corresponding author Takafumi Toyohara.

## Author contributions

T Abe, EK, and T Toyohara performed conceptualization. YO, YA, RM, MA, T Tanaka, T Abe, EK, and T Toyohara developed methodology. YO, YL, NS, TK, YT, YY, T Tominari, ST, YS, HK, SS, KI, CS, AK, T Akiyama, SM, and T Toyohara performed investigation. TS contributed to methodology and investigation (metabolomics). YO, YL, EK, and T Toyohara wrote the original draft of the manuscript. NS, MA, EK, T Abe, and T Toyohara reviewed and edited the manuscript. YA, RM, MA, HO, T Abe, EK, and T Toyohara acquired funding. MA, HO, T Tanaka, T Abe, EK, and T Toyohara supervised the study.

## Declaration of generative AI and AI-assisted technologies

Generative AI was used solely to assist in creating the schematic illustrations, icons, and initial visual layout of the graphical abstract. Full details are provided in the Acknowledgments section.

## Conflict of interest

The authors have declared that no conflict of interest exists.

## Funding support

- Japan Society for the Promotion of Science KAKENHI grants 16H04800, 21H05255, 24H00564, 24K22012, 26H00967, and JP24687027 (to EK); 18H02822, 20K20604, and 21H02932 (to T Abe); 21K08245 and 24K11379 (to T Toyohara); and JP21H05278 and JP22K15736 (to HO).
- Japan Agency for Medical Research and Development (AMED) grants 20ek0210133h0001, 20ak0101127h0001, 22ek0210168h0001, 23ek0210168h0001, and 24fk0108655h0003 (to T Abe); 22ek0210168h0001 (to T Toyohara); and JP23bm1423002 and JP23bm1123046 (to HO).
- AMED Moonshot Research and Development Program grants 21zf0127001h0001, 22zf0127001h0002, 23zf0127001h0003, 24zf0127001h0004, and 25zf0127001h1105 (to T Abe).

## Supplementary Material

Supplemental data

Supplemental video 1

Supplemental video 2

Supplemental video 3

Supporting data values

## Figures and Tables

**Table 1:**
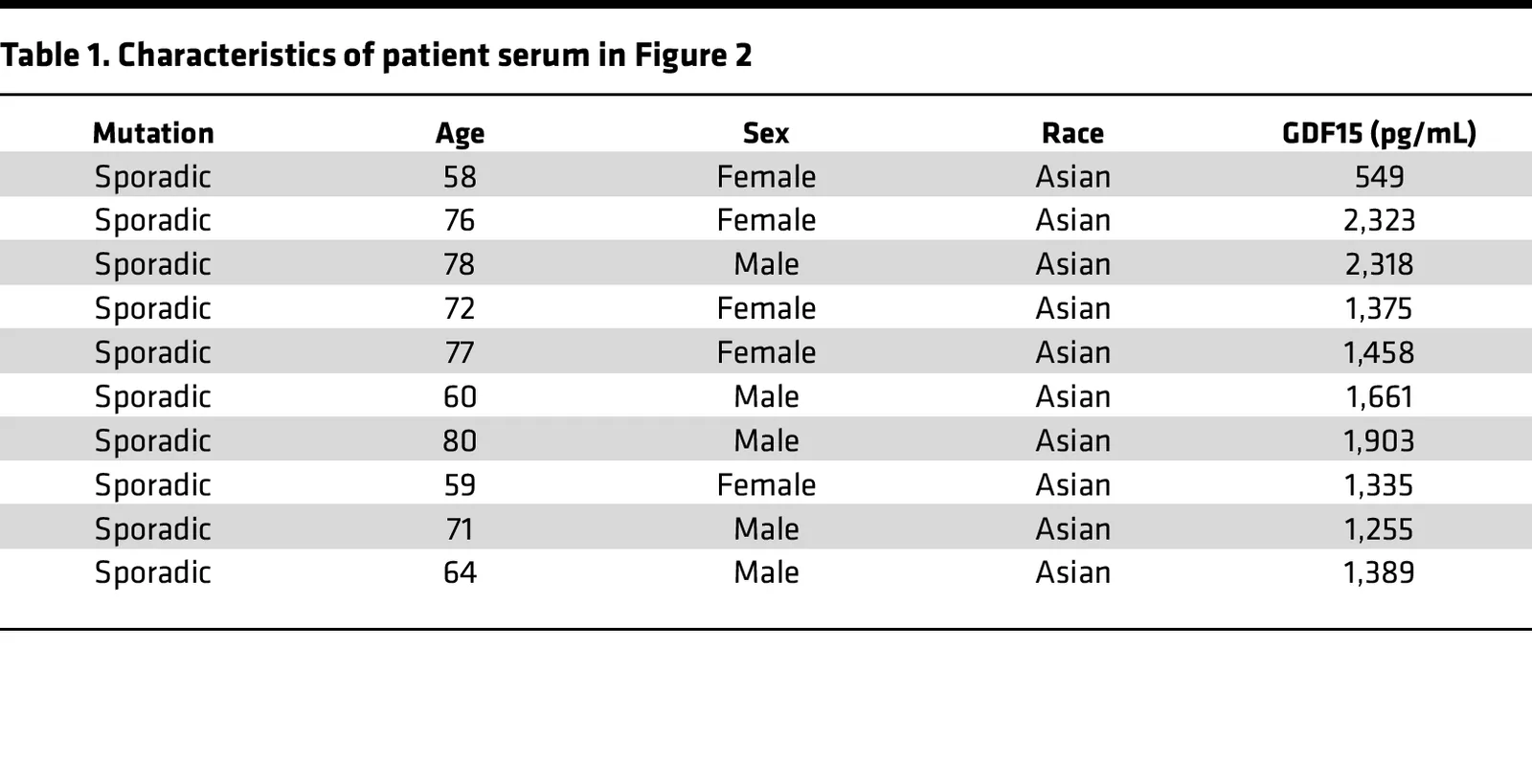
Characteristics of patient serum in Figure 2

**Table 2:**
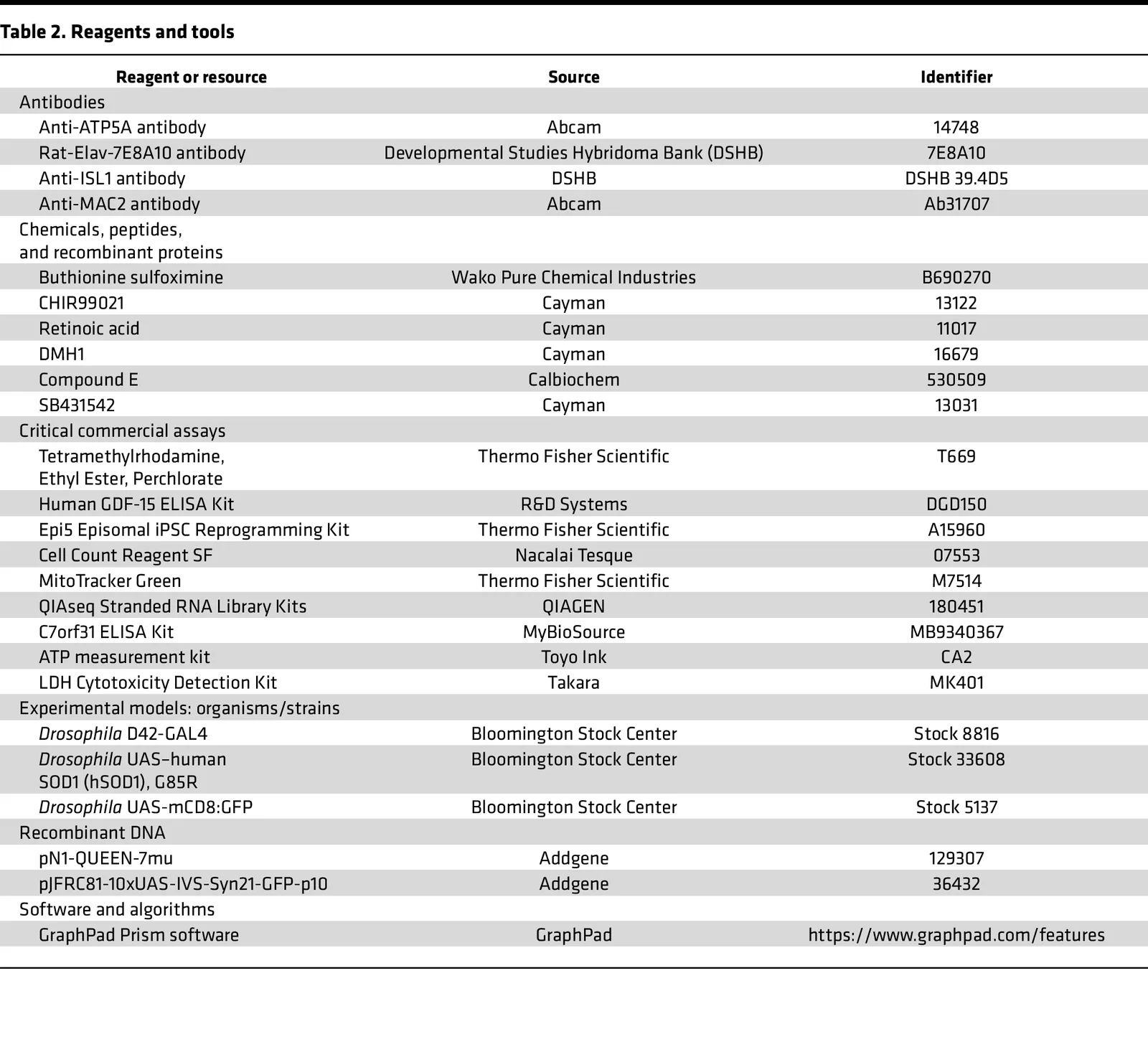
Reagents and tools

**Table 3:**
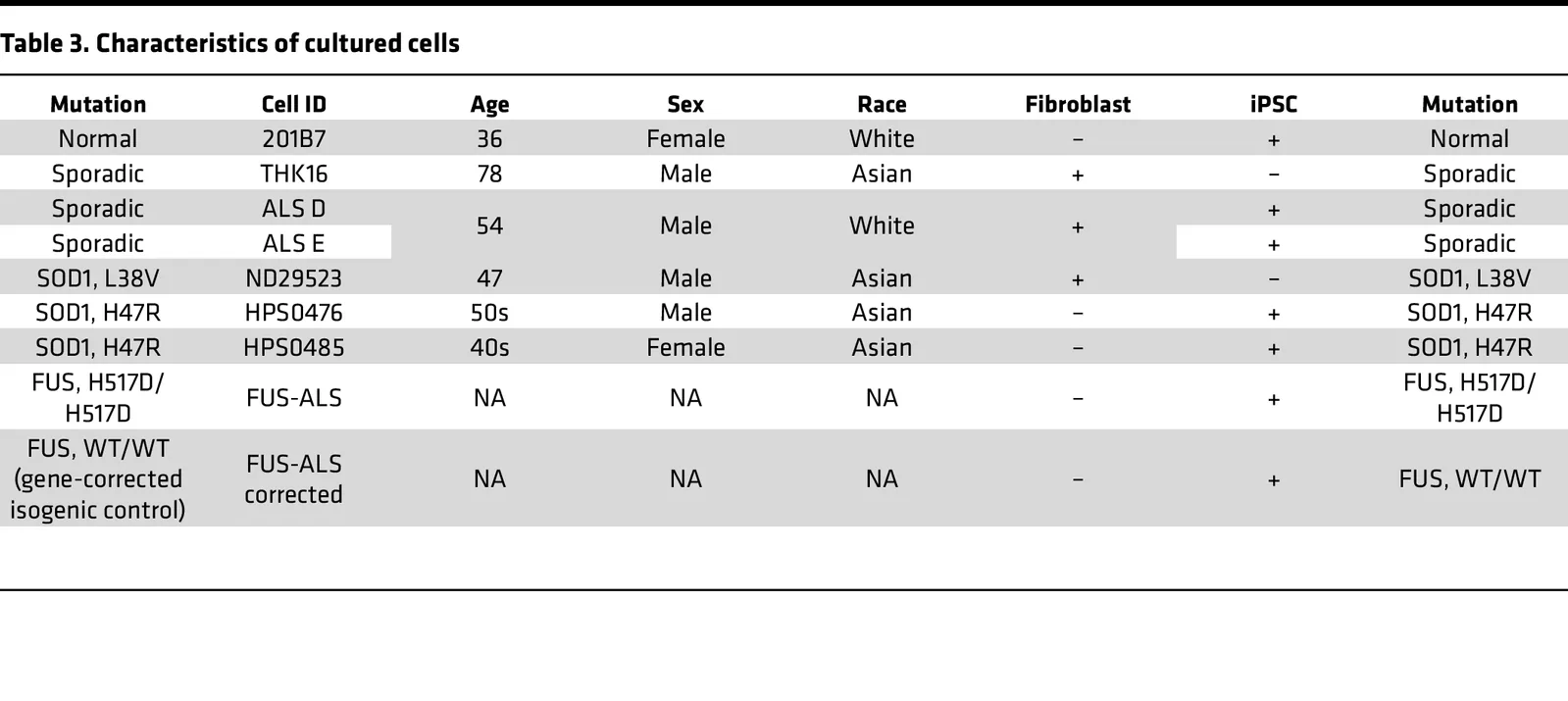
Characteristics of cultured cells

## Version 1 09/22/2026

Electronic publication
