# Supplementary material for: Mitochonic acid 5 alleviates amyotrophic lateral sclerosis phenotypes via mitochondrial augmentation
Source: JCI Insight. 2026 Sep 22;11(18):e200761. doi: 10.1172/jci.insight.200761 (PMC13596712; doi:10.1172/jci.insight.200761)
Supplement: Supplementary file 1 [file jciinsight-11-200761-s212.pdf]

Supplemental Figure 1

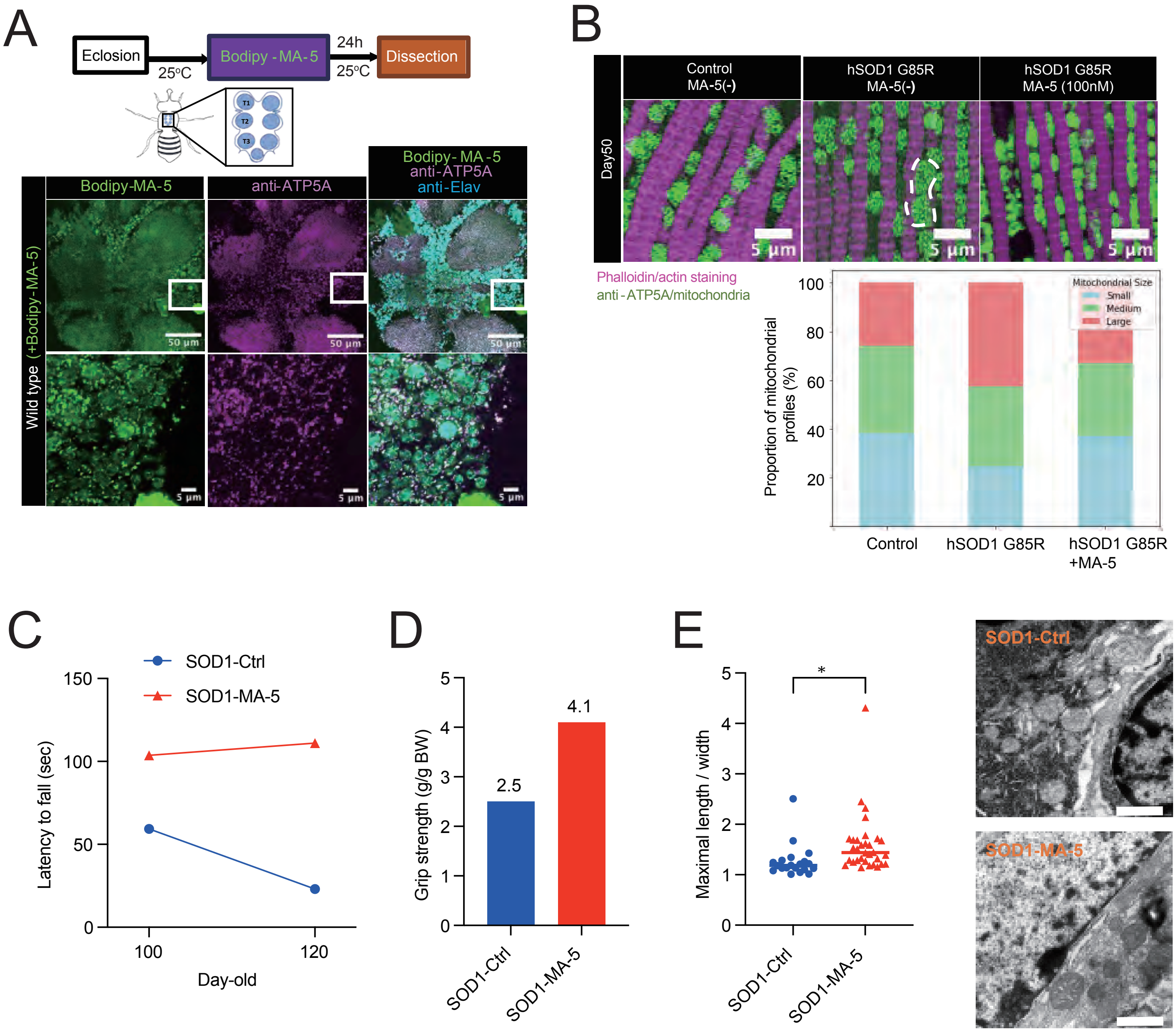

Supplemental Figure 1. Delivery of MA-5 and its effect on muscular cells in Drosophila and ALS model mice.

(A) Distribution of Bodipy-MA-5 in Drosophila neural tissues. Markers: ATP5A (mitochondrial marker) and Elav (neural marker). (B) Mitochondrial size in Drosophila muscle tissue (Small: < 2.56 μm², Medium: 2.56 - 5.31 μm², Large > 5.31μm²). Actin fibers were stained with phalloidin (actin marker). (C) Rotarod performance in SOD1^{G93A} ALS model mice treated with or without MA-5 (n = 1 per group). (D) Grip strength test in SOD1^{G93A} ALS model mice treated with or without MA-5 (n = 1 per group). (E) Mitochondrial morphology was assessed by calculating the aspect ratio, defined as the ratio of maximal length to width, in SOD1^{G93A} ALS model mice treated with or without MA-5. A total of 20 mitochondria from the control mouse and 32 mitochondria from the MA-5-treated mouse were analyzed. Scale bars: 1μm. Data in (B) were analyzed using chi-square test. Control vs hSOD1 G85R ($p < 0.001$) and hSOD1 G85R vs hSOD1 G85R + MA-5 ($p = 0.05$). Data are represented as mean ± SE. *p < 0.05

Supplemental Figure 2

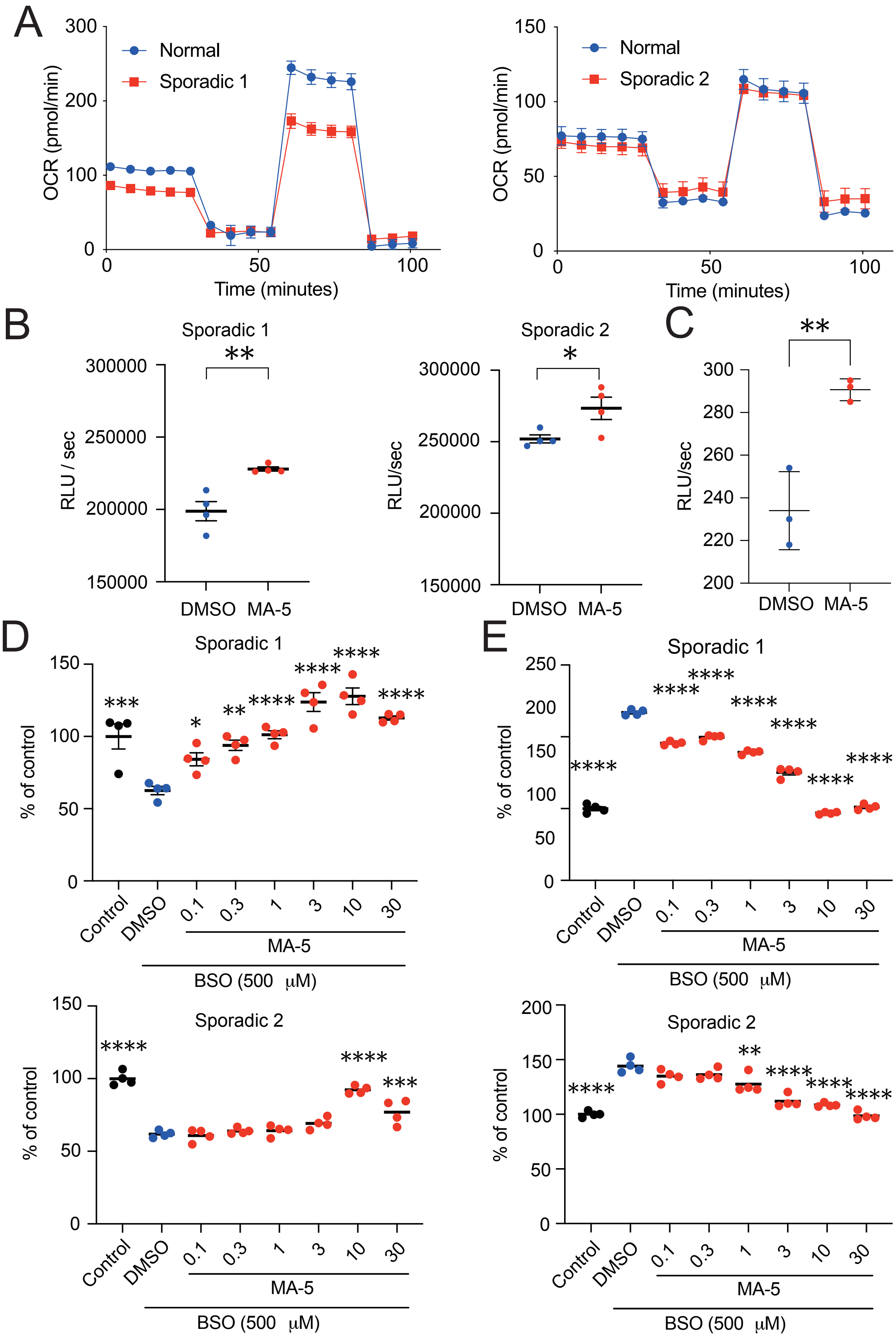

Supplemental Figure 2. MA-5 upregulated ATP production and protected against oxidative stress in fibroblasts derived from patients with sporadic ALS. (A) Mitochondrial function in fibroblasts from patients with sporadic ALS and healthy controls measured using a Flux analyzer (N= 4 per group). (B) ATP production measured at 6 h after DMSO or MA-5 treatment (10 μM) in fibroblasts derived from patients with sporadic ALS (n = 4). RLU: relative light unit. (C) ATP production measured at 20 min after DMSO or MA-5 treatment (10 μM) in mitochondria isolated from sporadic 1 patient fibroblast (n = 3). (D and E) Protection against oxidative stress by MA-5 in fibroblasts derived from patients with sporadic ALS. (D) Cell viability was measured using the MTT assay. (E) Cell damage was measured using the LDH assay. MA-5 was added at 0.1, 0.3, 1, 3, 10, and 30 μM (n = 4). Data are represented as mean ± SE. *$p < 0.05$, **$p < 0.01$, ***$p < 0.001$, and ****$p < 0.0001$. (B) and (C) were analyzed by t-test, while (D) and (E) were analyzed using one-way ANOVA and compared with DMSO.

Supplemental Figure 3

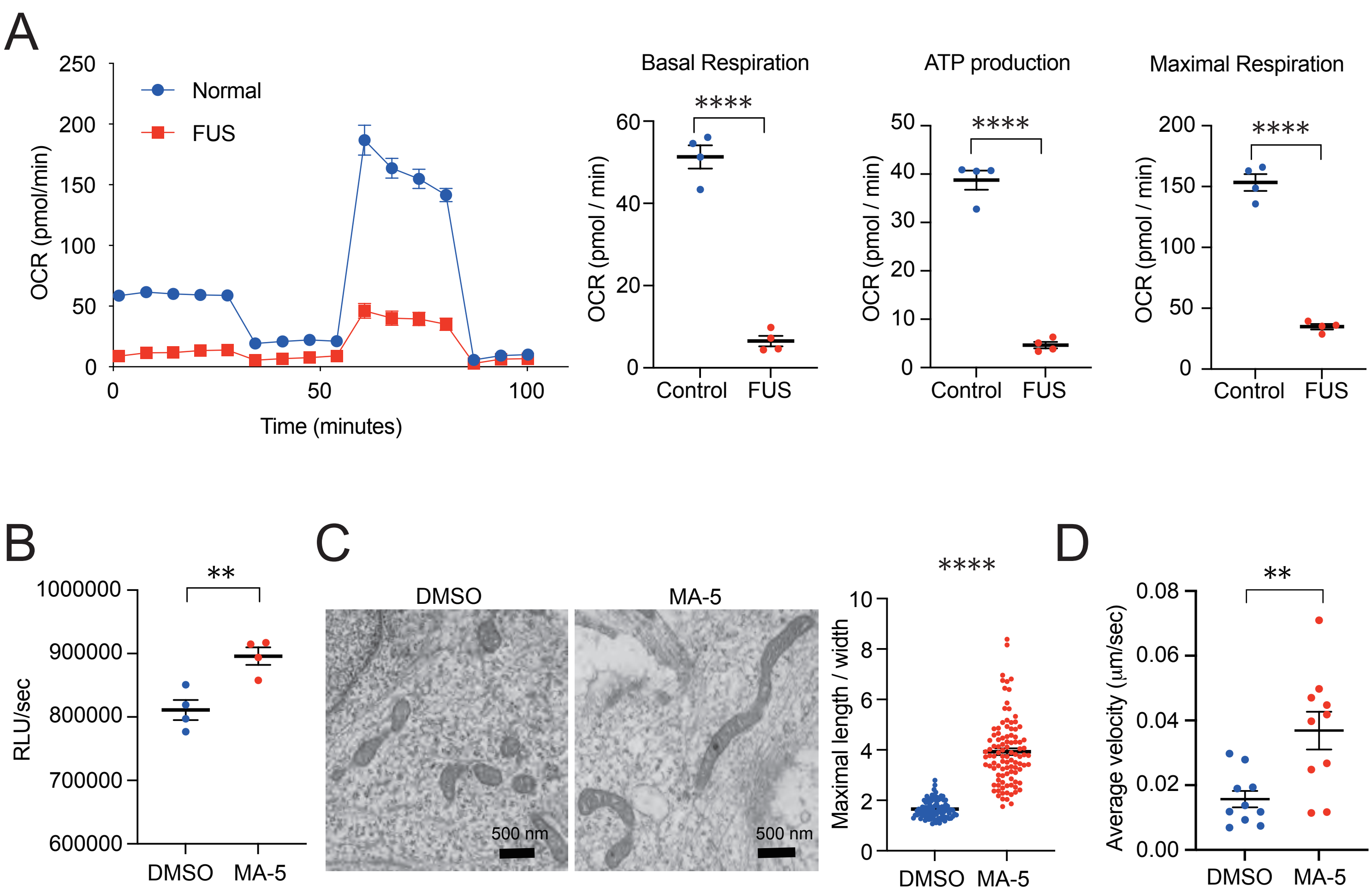

Supplemental Figure 3. MA-5 reversed mitochondrial dysfunction and structural abnormalities in motor neurons derived from FUS-ALS iPSCs.

(A) A Flux analyzer was used to assess mitochondrial function in motor neurons derived from FUS-ALS-iPSCs and healthy controls (n = 4 per group). (B) ATP production measured at 6 hours after DMSO or MA-5 treatment (100 nM) in motor neurons from FUS-ALS-iPSCs (n = 4 per group). RLU: relative light unit. (C) Mitochondrial malformation was assessed by calculating the ratio of maximal length to width in motor neurons derived from FUS-ALS iPSCs. Measurements were performed on 100 mitochondria, with or without MA-5 treatment (100 nM). (D) Mitochondrial movement velocity in motor neurons derived from FUS-ALS-iPSCs (n = 10), with or without MA-5 treatment (100 nM).

Data are represented as mean ± SE. **p < 0.01 and ****p < 0.0001. (A–D) were analyzed using t-test.

Supplemental Figure 4

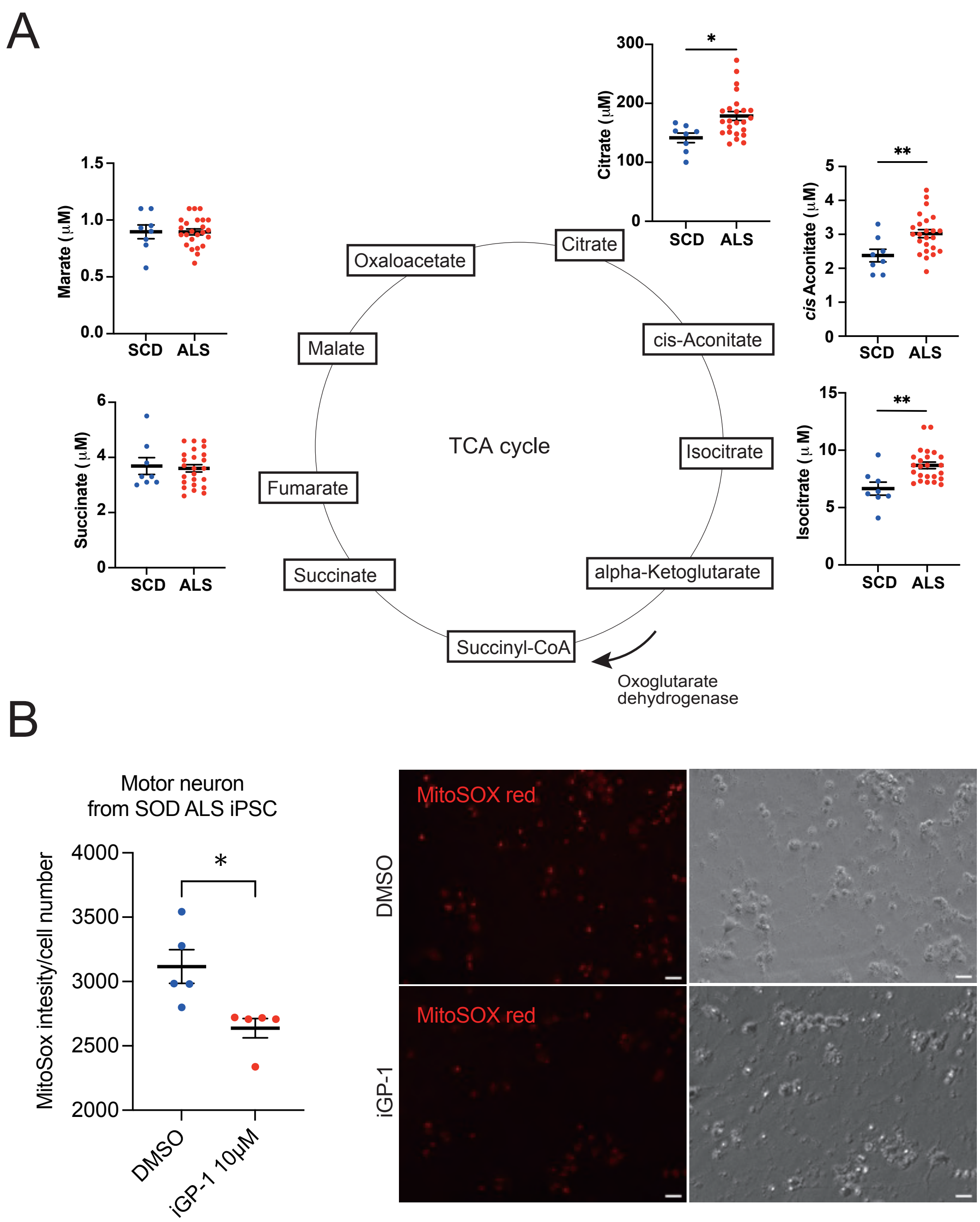

Supplemental Figure 4. Cerebrospinal fluid metabolites in patients with ALS and inhibition of the glycerophosphate shuttle in iPSC-derived motor neurons.

(A) Metabolites in cerebrospinal fluid from patients with ALS (n = 24) and patients with spinocerebellar degeneration (n = 8) were analyzed. (B) Mitochondrial ROS production in SOD1-ALS-iPSCs-derived motor neurons treated with or without iGP-1 (n = 5). Scale bars: 20 μm.
